# Between desire and rape – narratives about being intimate partners and becoming pregnant in a violent relationship

**DOI:** 10.3402/gha.v6i0.20984

**Published:** 2013-12-05

**Authors:** Kerstin Edin, Bo Nilsson

**Affiliations:** 1 Epidemiology and Global Health, Umeå University, Umeå, Sweden; 2 Department of Nursing, Umeå University, Umeå, Sweden; 3 Umeå Centre of Gender Studies, Umeå University, Umeå, Sweden; 4 Culture and Media Studies, Umeå University, Umeå, Sweden

**Keywords:** spouse abuse, sexuality, pregnancy intention, gender identity, narratives, Sweden

## Abstract

**Background:**

Women subjected to intimate partner violence (IPV) experience different forms of abuse. Sexual violence is often under-reported because physically abused women, in particular, might see forced sex as an obligatory part of the sexual interplay. Accordingly, abused women have less sexual autonomy and experience unplanned pregnancies more often than other women.

**Objective:**

To describe and analyse nine Swedish women's retrospective stories about IPV with a focus on power and coping strategies as intimate partners, particularly regarding experiences of sex, contraception, and becoming pregnant.

**Design:**

Nine qualitative interviews were carried out with women who had been subjected to very severe violence in their intimate relationships and during at least one pregnancy. The stories were analysed using ‘Narrative method’ with the emphasis on the women's lived experiences.

**Results:**

Despite the violence and many contradictory and ambivalent feelings, two of the women described having sex as desirable, reciprocal and as a respite from the rest of the relationship. The other seven women gave a negative and totally different picture, and they viewed sex either as obligatory or as a necessity to prevent or soothe aggression or referred to it as rape and as something that was physically forced upon them. The women's descriptions of their pregnancies ranged from being carefully planned and mostly wanted to completely unwelcome and including flawed contraceptive efforts with subsequent abortions.

**Conclusions:**

Women subjected to IPV have diverse and complex experiences that have effects on all parts of the relationship. Intimacy might for some turn into force and rape, but for others sex does not necessarily exclude pleasure and desire and can be a haven of rest from an otherwise violent relationship. Accordingly, women may tell stories that differ from the ones expected as ‘the typical abuse story’, and this complexity needs to be recognized and dealt with when women seek healthcare, especially concerning contraceptives, abortions, and pregnancies.

In an international perspective, from one-third to as many as half of all women who were specifically asked say that they have been subjected to sexual violence (1, 2). Women who report sexual abuse most often accuse someone they know, especially their intimate partner (3, 4). The impact of sexual violence is most likely more traumatic for an intimate partner compared to a non-partner (5). However, women often fail to label themselves as being raped in an intimate relationship and might even see forced sex as an obligatory part of the relationship (6). Consequently, for many different reasons, sexual violence is highly under-reported to police and healthcare officials (3).

Women subjected to violence have unplanned pregnancies more often than other women (7, 8). This is a result of forced sex that often occurs in combination with an inability to practice birth control (9–11). For unintended pregnancies ending in a live birth, the risks are significantly increased for having a pre-term delivery and of having a baby with low birth weight (12). Thus, intimate partner violence (IPV) might have synergistic effects that lead to increased risk for adverse pregnancy outcomes (10).

Instead of attempting to overcome the barriers to seeking help, abused women in a relationship with ongoing violence often choose to endure their situation because they fear the unknown changes and subsequent consequences of seeking help (13). Accordingly, when abused women seek healthcare for diverse reasons, they often do not disclose the violence of their own accord, but still they generally appreciate and support the idea of being asked (14, 15). However, even if women honestly respond to direct questions, many do not trust authorities to interfere and, therefore, wish for no further support (16).

In order to deal with women who are subjected to IPV, there is much that is needed to be understood about their situation because abused women live in complex and often very bonding relationships (17). The caregivers’ behaviour and their methods for building trust are, therefore, of vital importance if their relationship with their patient is to have a successful outcome (18–21). The barriers are two-sided and while the women keep their distance afraid of what will happen if they disclose their situation, healthcare personnel might not manage to get close to their patient because of the sensitivity of the topic (21, 22), especially if the woman is pregnant.

This study is about women in Sweden who became pregnant in a violent relationship. Gender equality has been an important political issue in Sweden, but despite the attention paid to this issue, the prevalence of violence during pregnancy is in line with what has been reported from the United States, Australia, and Canada. Furthermore, abuse can occur regardless of the woman's socio-economic status, and women who are exposed to severe violence before pregnancy usually experience violence during pregnancy as well. This perhaps indicates the importance of screening for abuse in Sweden (23). However, the term *IPV screening* is problematic (21, 24), and healthcare providers using streamlined screening questions together with their simplified understanding of IPV might not identify components such as women being forced into pregnancy (8). For several years, there has been an international perspective that requests a deepened understanding and more comprehensive view of the complex life situations of abused women (25, 26).

The overall aim of this paper is to describe and analyse nine Swedish women's narratives about their violent relationships and about having sex and becoming pregnant. The primary focus of the paper is on power and coping strategies, especially the complexities and contradictions within and between the stories.

## Methods

### Qualitative methods

We carried out interviews that aimed to acquire the broadest and deepest understanding of women's experience with IPV, and we used narrative methods for the analysis. The most weight was put on understanding the meaning of the women's lived experiences, including their own reflections and considerations, and less weight was placed on how the stories were told linguistically (27). The interviews were treated as dialogues that supported diverse beliefs and assumptions in order to create shared constructions of meaning between the interviewer and the informants (28, 29). Such constructed knowledge is never constant but is always changing as a result of interpersonal, social, cultural, and political influences. This kind of knowledge is also often incomplete because the person providing the information has the power to decide what information is included or excluded (30). Accordingly, the narratives in this paper represent the stories that the women chose to give at that particular time and place (31, 32).

### Subjects, interviews, and analyses

We contacted the coordinators of shelters and a women's crisis centre in regard to this study and asked for their help in identifying women who earlier had received support and counselling from them and who matched the specific criterion for inclusion. This criterion was having been subjected to violence by an intimate partner in a relationship in which they had become pregnant. Ten women were invited to be interviewed, but one subsequently decided not to participate and dropped out of the study. The nine remaining women were interviewed by the first author, three in 2001 and six in 2003, and each interview lasted for about two hours. An interview guide with open-ended questions was used, and some questions could be omitted or expanded upon and the exact wording and sequence could differ (28, 33). The interviews resulted in a large amount of data, and the selections presented here were chosen based on their applicability to the main focus of the paper (see also 21).

The women's narratives about the violence were in general very detailed, lively, and dramatic. The interviewer was not only a partner in the dialogue, but often found herself attending a performance that through the details, laughter, tears, different voices, metaphors, and quotes transported us both back to the place and time where the events had taken place. Nevertheless, repressive mechanisms were apparently used by some women. They were often hesitant or had difficulties in recounting the memories and provided only partial and patchy recollections when it came to some especially tough details.

The interviewer, a mother with a background as an RN midwife, appeared to build a trusting relationship that enabled the women to include sensitive issues while adding statements like *as you know*. One woman cried a lot during the interview, but felt much relieved to have told certain parts of her story for the very first time, even if the subject matter was extremely sensitive. All of the women gave the impression that they found it of great value to share their stories. To assure support and someone to talk to, all of them received the interviewer's contact information after the interviews. Moreover, all of the women, because of ongoing or previous contacts with the shelters/centres, had a familiar helpline if needed.

The interviewer took notes and performed preliminary analyses for each interview, and the recordings were transcribed verbatim and coded into codes and categories to interpret the probable meaning as a whole and to identify crucial tracks to provide an analytical framework with narrative themes in which to present the stories (34, 28). We provide one example of the coding process in Table 1. Both of the authors took part in processing the entire interview material, and the initial analysis made by the first author (KEE) was discussed and interpreted by both authors together. The narrative themes could not be presented as just one coherent and typical story because all of the stories contained differences, contrasts, and contradictions (29). Instead, the different voices are either intertwined into representative narratives or written as individual stories (28). Direct quotes from the women are written in italics with the purpose of emphasizing or providing examples of selected narrative themes.

**Table 1:** Examples of selected codes and categories building up a narrative theme

Selected codes	Categories	Narrative theme
Adverse feelings, forced, all the time, spoiled her IUD contraception, sexual violence, abortions, never wanted a child, not accepting it to happen again, wanted to leave, he needed to find another woman	Forced, did not want to, never, wanted to escape from him, had contraception	*I did not want to become a parent*

### Ethics

The research process aimed to maintain ethical considerations regarding confidentiality, safety, and support by following WHO recommendations (35). The study was approved by the local Ethics Committee of the Faculty of Medicine, Umeå University.

## Findings

The nine women were approximately between 31 and 55 years old at the time of the interview. They had varied educational backgrounds and socio-economic status and different job positions; two were on sick leave. The women had a total of 25 children from 20 long-term non-violent and violent relationships, and five women had been married to their violent partner. They told stories about how they had been subjected to different forms of brutal physical and/or sexual violence and/or threats of severe violence during a total of 14 pregnancies. In five of the cases, the pregnancy had started or restarted the violence, and for the others the pregnancy occurred during ongoing violence that either became more aggressive or maintained its current level. Generally, the women expressed feelings of having been unable to escape from a situation that was chaotic and very, very strange. Two women left their violent partners while being pregnant because of life-threatening violence, and the others left when their last child was still a toddler. Even if some of the women had tried to recommence their relationships, all of them appeared to have left their partners permanently (from about 8 months up to 16 years ago) at the time of the interview (*cf*. 21).

The following presentation deals with the women's narrated experiences of their intimate relationships and is divided into the following three sections: the relationship, having sex, and becoming pregnant and includes eight narrative themes: ‘Like in a prison’; ‘As if they were worthless’; ‘To always be on one's guard’; ‘Sex as violence and as a part of the violent relationship’; ‘Sex as the only thing that actually worked’; ‘I did not want to become a parent’; ‘So I became pregnant’; and ‘A wanted child’.

### The relationship

The interviewed women portrayed their partners as tormentors and called them a *devil* or described them as *Jekyll and Hyde* as they changed from good to bad. This *Jekyll* was present in the narratives as the charming and nice man that they had fallen in love with. Some nurtured the hope of change and expressed wishful thinking that the relationship could return to how it was when they had first met.^1^

#### Like in a prison

The lives of these women became very restricted, and they were isolated from their former friends. Often the women did not even understand why they had to obey and follow certain strange rules or else they would be punished. The feelings this caused were not just emotional but were converted into physical sensations such as feeling that they were being kept in a prison from which they had small chances of escaping. Sometimes they had no possibility to use the telephone, and the car keys were hidden away or the car was never left for them to use.

(He) shut me in, kind of […], was not allowed to borrow the car, and took it to work even if he did not need to, just so that I would not be able to go anywhere. (W1)

The women also described how their partners withheld or controlled money. Two women were entirely financially dependent on the man with absolutely no money of their own, and others did not have enough money for food or they needed to borrow money to go shopping.

#### As if they were worthless

The women brought up many examples of how they were subjected to all kinds of severe physical and sexual violence and threats of such violence from their partners. The women also described psychological violence such as limitations, prohibitions, harassment, isolation, depreciation, subordination, manipulation, destruction of dear belongings, threats of losing custody of their children, and how the partner showed jealousy or tried to make the women jealous. The partners often used foul language, sometimes even in front of others, and would often tell the women to shut up and/or roar out such things as *bloody whore*. The women were criticized, humiliated, and felt worthless as a person.

Yes, he thought that almost everything I did was wrong […] yes I became smaller and smaller, you see… (W3)

The women were the recipients of so many negative statements in so many different ways, and felt depreciated as both partners and mothers, that they had lost almost all sense of self-worth (*cf*. 21).

#### To always be on one's guard

For all the women living in a relationship with ongoing violence, the constant anxiety of further violence seemed to be the worst part. The violence was like an omnipresent shadow and tension in the relationship even during days when nothing happened. The outbursts were often unpredictable, but sometimes there were certain *signals* indicating when it was important to be more watchful and when the woman might be able to keep calm and make her partner listen to reason. To further avoid the violence that was often felt to be life threatening, the women learned to get out of the way, to adapt, to behave in a certain way, and not to give free rein to their own personalities and to obey him unquestionably. However, all of this was often fruitless because the partners looked for reasons to get angry and to start conflicts, and then it was difficult to stop them. Thus, the women almost never felt safe.

… you don't know how to act… you can walk on tiptoes, but you know it makes no difference whatever you do, however you try to fit in with him, you never know how he will react…(W7)

One woman recalled an alternative way of dealing with her partner's violence. She sometimes reacted to his signals with a fit of anger of her own, such as throwing things at him, as a way of speeding up his violent outbursts. She then knew that the violence would be over soon and that this would be followed by a period of calm.

A variety of strategies were described as ways to handle the everyday struggles, and one woman said that she did everything to focus her mind in a certain way, to pretend that her strange life situation did not exist. Sometimes certain parts of the violent events were repressed and just fragmented memories remained such as *it happened in the living room* and *I was lying on the floor* and the details of the violence were forgotten.

### Having sex

#### Sex as violence and as a part of the abusive relationship

The women described how their lovers had turned into perpetrators and some also described how sex had changed from a matter of desire to one of aversion. In seven of the nine narratives, sex was described as something that had become more and more negative. One of the women described the situation as follows.

Yes, if I just get this done so I can avoid it later, then I will be let off that nagging […] but then finally it became harder and harder for the body[…] it did not want to, it did not work…(W4)

One woman told about her partner wanting to have sex after the violence as a way to say he was sorry, but she refused. Some women were physically forced to have sex while others viewed it as a necessity, something they had to take part in without any pleasure for themselves. Thus, sex was often used to avoid or postpone violence and even to calm him down after a violent act. To refuse sex was a risk, so even if the women did not have any desire for it, having sex could be used as a strategy to safeguard themselves and their children from something that might be even worse. One woman (W9) could not understand how her partner first could tell her how *fat, ugly, and disgusting she was, and, ugh, he did not even want to touch her with a bargepole* and then he suddenly wanted sex. It seemed to her as if men in general believe that sex and love are two totally separate matters. However, it was best just to agree to it, otherwise he would spank her. But she felt empty and disgusted, and as a way to endure she did not interact at all.

Yes, I did nothing and then I got to hear that I was inanimate, I was equivalent to sticking it into the mattress … (W9)

In general, to refuse sex or not was like choosing between the plague and cholera, and sex without warm feelings was nothing the women could get used to but rather it became worse and worse. One partner always wanted sex, and although the woman tried everything to escape, she felt she often just had to give in to his demands.

… I was so terribly disgusted with him. And then he said afterwards that ‘It felt just as if I had raped you.’ I thought to myself, ‘Can't you understand, that is precisely what you did except that I didn't lash out or fight’. (W7)

One of the women lived in a relationship where she was forced to have sex as often as three times every night. If she refused, he would discipline her by using violence such as pinching or kicking her out of the bed or he behaved badly the next day. He felt that if she did not allow him sex she did not have the right to sleep there, it was his bed. This even continued during pregnancy and immediately after she came home with the baby from the maternity ward (they were even discharged early from the hospital because he wanted her to come home).

Just for this reason, because if he was not allowed to sleep (have sex) with me three times, then it became a downright rape, yes the first…..is also a rape, you see… but… (W3)

One of the women told about how her partner, in the midst of a sexual act that had begun in a nice way, could just change it into an assault. Maybe she said it hurt and that resistance turned him on. She also told about being raped when she was at the end of one of her pregnancies, she was having a shower and he came in to her.

Then I chose to do nothing, that time I did absolutely nothing. I said I did not want to and then I just cried, then I did not do anything more. (W4)

For this woman, talking about the violence during pregnancy, and revealing the sexual aspects of it in particular, was like dragging the whole pregnancy through the dirt, and she did not even want to think about her children finding out about it.

#### Sex as the only thing that actually worked

Two of the nine women were able to maintain a positive give-and-take intimacy and were able to keep their sexual life separate from the violent relationship. It was like having two separate relationships with the same man. When having sex, the partners were suave, took an interest in them, and saw them as worthy individuals.

Our sex life functioned well; I have never felt threatened sexually, no, not at all. There he was like another person, then he was soft, it felt like I became close to him…no, he was never, he has never been thre….threatening in that way… (W1)

One of these two women said that when they were having sex, the partner was so aroused that he gave her the attention she never got otherwise, although she admitted that this was less pronounced during the really bad periods. However, sex was generally a relief for these two women, a breathing space, like it happened with him as another person, a gentle person. This made it possible for the women to feel closeness and intimacy.

### Becoming pregnant

#### I did not want to become a parent

Some narratives included a reluctance to become parents, and three pregnancies occurred despite a strong desire to avoid becoming pregnant and to enter parenthood. One woman had been living in a relationship with continuous sexual violence, and with a potential child in mind these are the thoughts she had at the time.

… So I sort of hit out ‘don't want to, don't want to, don't want to’ since he… I was on the pill, then suddenly they were just lost, you know, none left. When I had a loop (IUD) then he hurt me so terribly bad when I had that, so I had to have it removed because I could not manage … since he wanted me to become pregnant. And definitely, then when he found out that I had an abortion a new trial started, ‘how could I be so mean to him?’. (W3)

This woman knew that becoming a mother meant that she would have to take full responsibility for the child and she did not see herself as a good mother, but she felt forced to have a child. Finally, after she had used contraceptives, had abortions, and desperately tried to protect herself from her partner coercing her into pregnancy, she gave up her opposition. The pregnancy did not give her any respite; the partner continued the sexual violence just as before.

#### So I became pregnant

Most of the pregnancies described were unplanned and the women gave the impression of having a rather ambivalent stance or a laissez-faire attitude towards the pregnancies. One woman was reluctant to take contraceptive pills even though she had gone through an earlier pregnancy that had started the violence, and one did not mention using any contraceptives despite experiencing escalating violence.

… No, it was not planned. Actually I had these golden pills, since I took no contraceptive pills or anything and we should protect ourselves then, but at that point it was not so much of that then … (W4)

Regarding these unintended pregnancies, the women seemed to have left things more or less to chance, used no or ineffective protection, and had not really thought much about the possibility of becoming pregnant. When one of them told her partner that she was pregnant, he wanted her to have an abortion but she refused. From these women's point of view, sex was mainly something that was neither anticipated or planned for, but rather often happened in spite of themselves and was associated with very tense and violent circumstances. Consequently, contraception was not a natural subject of conversation and was not something that was easily negotiated.

#### A wanted child

Some women talked about a joint or individual desire to have a child. For two women, having a baby was a decision they made themselves and was something they really wanted; the child was supposed to be a love child (these two women were not the same two women who described sex as a positive experience in the previous section about having sex).

But then if one should talk about the pregnancy now, well, there was nothing happy about it, you know, unfortunately it was not so. You see, this was supposed to be a love child … (W6)

The women had this desire for a child despite their complicated relationships with steep ups and downs and where they had broken it off with their partner and then been reunited. One of the women even suffered ongoing severe violence while planning for a baby.

I actually had Olivia (not her real name) because I really wanted to. I really gave 100% because I really believed in our relationship. I really believed that … this (the violence) is not true, something like this can't happen. I thought I really was able to overcome this, I really believed that … (W7)

These two women did not actually believe that a baby would eliminate the negative aspects of their relationships. Instead, having a baby seemed to sanction the positive aspects of the relationship, their long-standing friendship, passion, love, strong feelings, and a belief that they were meant for each other.

## Discussion

According to the interviews, besides the threats and the physical and sexual violence, their partners also made the women feel criticized, humiliated, and worthless. The women also felt very restricted in their relationships, isolated from friends, and limited in their lives outside the household. Despite this and many contradictory and ambivalent feelings, two of the women in this study described having sex as a reciprocal desire and as a respite from the rest of the relationship, but the others gave a negative and totally different picture. These women viewed sex as either obligatory or as a necessity to prevent or soothe aggression, or brought it up as rape and something that was physically forced upon them. The women's descriptions of their pregnancies ranged from being carefully planned and mostly wanted to being completely unwelcome and as occurring after flawed contraceptive efforts and previous abortions.

### Methodological considerations

There might be some uncertainty regarding the selection of women because the selection was carried out by the coordinators of the women's crisis centres where all of the women had sought help at some point. Thus, the coordinators could have used some additional inclusion/exclusion criteria besides the written instructions from the interviewer. Moreover, the women told retrospective stories, some of which had occurred several years prior to the interview. However, this is not believed to be a severe limitation because research has found that memories of harrowing events remain rather stable over long periods of time (36, 37). While the first author did a detailed analysis shortly after the interviews that resulted in narrative themes, some years have passed since then, and that might be viewed as a limit. Conversely, we believe that both authors’ later discussions and comparisons between the initial narrative themes and the entire interview transcriptions have been important and might even strengthen the final analytical interpretations and the trustworthiness of the study.

Our narrative study gives a deepened understanding of abused women's life situations (*cf*. 21) and is also related to studies about complexity, paradoxes and opposite discourses in violent relationships (*cf*. 17, 38, 39). However, we have not found other studies about IPV in the range between the extremes of desire and rape and wanted versus unwanted pregnancies. We believe that the results of our study are transferable knowledge and can add useful insight into the complexity and manifold nature of abused women's intimate relationships.

### Analyses

During the analysis, it became obvious that in their narratives the nine women described (directly or indirectly) different power strategies used by their partners and different coping strategies that they themselves used to deal with their violent relationships (*cf*. 21). Furthermore, the narratives reported on turning points such as becoming pregnant and ‘breathing spaces’. The latter refers to a tendency in the narratives to keep positive aspects of the relationships separate from the negative aspects, which we analysed as a form of compartmentalization. Accordingly, the following discussion is divided into the following four sections: (1) Power strategies, (2) Coping strategies, (3) Turning points, and (4) Compartmentalization.

### Perpetrator power strategies

A significant amount of literature on IPV is related to power strategies and coping strategies. Power strategies are often categorized in terms of physical, sexual, and psychological violence (*cf*. 39). Male dominance, power, and control are major explanations that are given for violence and IPV (1, 40–45). Sexual violence is often considered the ultimate form of power and control (40), not least in relation to aspects of the relationship dealing with sexuality and fertility (46, 47). Furthermore, ‘sexual assault perpetrated by an intimate partner may be especially traumatic’ (5).

In this study, violence was a regular occurrence in the relationship and different power strategies were used by the male batterers. The women were often beaten or stabbed in addition to being subdued, harassed, isolated, and emotionally manipulated. Another recurring male power strategy was the use of undesired and forced sex, which was described by the women as repugnant and disgusting but also as an inescapable part of the relationship and the violence or as something that could be used to calm the partner down and even to prevent violence or end it quickly (*cf*. 48). The women often said that they interpreted the sexual acts differently compared to their partners (*cf*. 49).

### Coping strategies

The power strategies used by the men in the relationships did not result in a totally passive life for the women. Instead the women used different strategies to cope with the violence and to overcome their situation.

Coping strategies aim to preserve physical and psychological well-being in situations of stress and have been categorized in different ways. It is common to distinguish between problem-focused versus emotion-focused coping and between engagement versus disengagement. Whereas problem-focused strategies attempt to change the problem that causes the distress, emotion-focused strategies attempt to deal with emotional responses to the problem (50). A point of departure for this study is the index of different IPV coping strategies that was developed by Goodman and colleagues (51). The authors distinguish among six different categories of strategies. First, women can use a ‘formal network’ in trying to get help and deal with the violence. Second, calling the police or filing criminal charges are examples of ‘legal’ strategies. ‘Safety planning’ is a third category, and it includes different activities aimed at reducing the violence, for example, hiding weapons. Fourth, by turning to ‘informal networks’, women seek help from family and friends. ‘Resistance’ refers to women actively trying to stop or reduce the violence. Sixth, by the use of ‘placating’ strategies, women intend to change the abuser's behaviour but not challenge his sense of control.

We have used this index to identify two recurring strategies. For example, some of the women used *placating strategies* when they tried to please their partner in different ways – for example, by allowing him sex even if she did not want it, as a way to temporarily stop the physical violence. The interviews also reflected *resistance strategies*. The women were critical, obstinate, spoke their mind, and made their own decisions. For example, one woman refused to have sex after a violent situation, and another used contraceptives against her partner's will. However, there were limitations to their strategies of resistance, and they usually failed to prevent further violence (*cf*. 48).

### Turning points

For some women in this study, becoming pregnant was an aggravating circumstance. The pregnancy was often described as a fateful moment (*cf*. 52) and as a special life experience of importance for both the men's power strategies and the women's responses (i.e. their coping strategies). Pregnancy can also be described as a turning point because it was in relation to it that the violence started, restarted, or escalated (*cf*. 21). With two exceptions, the pregnancy just happened or was unintended as a result of the failure or the counteracting of contraceptive efforts. This is in agreement with other research (53) and is explained by forced sex and the obstruction of fertility control (9–11). The most extreme example in this study was the partner who systematically raped the woman and foiled her use of contraception in order to coerce her into pregnancy (*cf*. 47).

Pregnancy was not only a turning point regarding the male batterers’ use of violence but was also an emotional or physical turning point for the women (*cf*. 49). Two women left their partners during their pregnancies because their situation had become life threatening (*cf*. 54), but the others did not. Pregnancy in a relationship with ongoing violence may bring with it additional dreams about idealized images of parenthood and expectations of support for the baby instead of becoming a single mother (55). This appeared to be the case when some women described having a baby as a ‘choice’. The motivation was not actually to stop the violence, but by becoming pregnant the women sought to maintain or emphasize the good parts of their relationships. Here romantic (and utopian) ideas of passion and long-standing friendship played a major part – ideas that often characterized the abused women's stories about the beginning of their relationships (*cf*. 56). Moreover, a woman subjected to violence might still feed, consciously or unconsciously, her ingrained ideas about the nuclear family in which becoming a mother and having children plays a central role (17).

### Compartmentalization

The narratives in this study were characterized by contradictions and complexities such as the description of the partner being a *Jekyll and Hyde* who alternated between being good and being bad (*cf*. 57). This cannot be fully understood through power strategies, coping strategies, or with reference to pregnancy as a turning point. This is especially the case when it comes to sexuality and the range between desire and rape. The most extreme example of this was the two women who reported that they had a good sex life despite living under violent circumstances. To be able to comprehend this, we have introduced another theoretical concept, compartmentalization. This refers to a mechanism with which people try to avoid cognitive dissonance, and conflicting values, interests, and emotions can be dealt with by keeping them separate. Positive compartmentalization – which is more common – occurs when an individual's positive self-aspects, attributes, and self-beliefs are important and accessible, and it often leads to a positive mood. Negative compartmentalization means that an individual's negative self-aspects, attributes, and self-beliefs are important and accessible, and it results in negative mood and low self-esteem ((58); *cf*. (59)). Compartmentalization has been described as a sign of poor self-cohesion (60), but also as a very effective way of organizing self-knowledge, especially if negative attributes can be avoided (61). Integration is in contrast to this type of organization of self-knowledge and is characterized by a blend of positive and negative self-ideas (62).

In our view, compartmentalization works not only on a cognitive level but also on a physical level. This means that an individual can keep different sets of actions separate. One physical behaviour can be related to positive self-aspects under one circumstance, but connected to negative self-aspects in another. In other words, compartmentalization also has a physical or bodily dimension.

By connecting sex with positive feelings, associations, and bodily experiences, and by separating it from the violence, two of the women could continue to enjoy their sex life and feel intimacy and closeness. It also appears that their partners did the same thing, and violence and other power strategies were often replaced with more gentle verbal and physical behaviours. This physical and two-way compartmentalization laid the foundation for a good sex life despite the violent circumstances of the relationship.

### Conclusions and implications for practice

The results in this study are in line with other studies showing that women subjected to IPV have diverse and complex experiences that affect all parts of the relationship (17, 38, 57, 63, 64). According to the narratives in our study, intimacy might turn into force and rape for some, but for others, sex does not necessarily exclude pleasure and desire and can be a haven of rest from an otherwise violent relationship. Accordingly, women may tell stories that differ from the ones expected as ‘the typical abuse story’, and this complexity needs to be recognized and dealt with when women seek healthcare, especially concerning contraceptives, abortions, and pregnancies (*cf*. 38). Thus, further support and training can help practitioners in meeting with patients or abused women (65, 66) and to ask the right questions about intimacy and contraceptive use. This may help them to differentiate healthy relationships from unhealthy ones, and this can be helpful also for both women who do and do not identify themselves as having relationship problems (8). Such counselling might give the healthcare provider information about inconsistency and coercion in the relationship and might provide women with an opportunity to disclose and give clues about ongoing IPV (8). It might also be important to take mechanisms of compartmentalization into consideration, especially in terms of a physical or bodily dimension. Abused women can, as was the case in this study, report on positive and enriching sexual experiences while at the same time living under violent circumstances. Such reports can be interpreted as signs of a rather healthy relationship, when they actually could be signs of the exact opposite.

## Footnotes

1 One woman, after the recorder was turned off, said she was still in love with this man, the one she once had wished for.
